# “They don’t care about deaf problems.” Children’s rights and emotional well-being

**DOI:** 10.1093/jdsade/enaf063

**Published:** 2025-10-21

**Authors:** Bronagh Byrne, Catherine B McNamee

**Affiliations:** School of Social Sciences, Education and Social Work, 20 College Green, Queen’s University Belfast, University Road, Belfast, BT7 1LN, Northern Ireland, United Kingdom; School of Social Sciences, Education and Social Work, 20 College Green, Queen’s University Belfast, University Road, Belfast, BT7 1LN, Northern Ireland, United Kingdom

## Abstract

This study uses a mixed-method sequential approach to explore the prevalence, perceptions and experiences of deaf children in relation to their emotional well-being in Northern Ireland. While there is a small body of research exploring the application of human rights treaties for deaf children, there is an absence of research that applies a rights-based framework to their emotional well-being. Drawing on findings from our study we argue that deaf children experience a lack of inclusive time and space(s) across their lives, and that they carry undue burden and responsibilities in implementing their own rights. We argue that these factors contribute to the higher prevalence of mental health conditions they experience compared to their hearing peers. We further examine the role of a rights framework as a facilitator to better well-being, drawing on children’s views on ways forward, arguing that effective implementation of rights is a precondition to positive well-being.

## Introduction

“Good”, “strong” or “positive” emotional well-being is a desirable and important outcome for all children and young people, including deaf children.^1^ The importance given to well-being is such that it has been included as one of the 2030 United Nations (UN) Sustainable Development Goals alongside good health (Sustainable Development Goal 3, UN, 2015), foregrounding the need to understand, and appropriately and effectively address, inequalities in well-being between and within population groups including for deaf children. Emotional well-being is a critical component of overall well-being alongside other domains such as physical, social, environmental and material well-being (Choi, 2018). It is important to note that emotional well-being can be impacted by and impact on other well-being domains. Increasing efforts to measure and research child well-being in recent years (Ben-Arieh et al., 2014; Choi, 2018) have primarily been in the form of surveys or well-being indices (see for example Ferro et al., 2022; Moore, 2020; Ravens-Sieberer et al., 2022) with an emerging body of work beginning to look at children’s qualitative experiences of their emotional well-being (Carrillo et al., 2021; Fattore et al., 2019; Fattore et al., 2007; Vujčić et al., 2019). However, such research has tended to focus on children as a homogenous group and less attention has been given to specific groups of children including deaf children. Moreover, research on this topic has frequently gathered data from the perspective of parents and teachers with fewer capturing the voices of children themselves.

Studies examining deaf children’s well-being have taken a multitude of theoretical approaches. Medical-pathology perspectives have been heavily criticised and long fallen out of favour among deaf studies scholars for their deficit framing and focus on “cure” of the individual towards hearing based norms (Kusters et al., 2017). More recent work grounded in deaf studies explores the role of structural, cultural and linguistic barriers as the focus on inhibiting inclusion, removing the burden on deaf individuals and placing the issue on the social world to provide an inclusive environment (Kusters et al., 2017).

We propose building on this by utilising a normative rights-based framework based on the United Nations Convention on the Rights of the Child (UNCRC, 1989) and the United Nations Convention on the Rights of Persons with Disabilities (UNCRPD, 2006). These are important since they establish a range of obligations on states that have ratified them, with 196 States parties having ratified the UNCRC, and 191 the UNCRPD. While human rights treaties are imperfect mechanisms, not least around their enforcement (Collingsworth, 2002), they provide a conceptual lens through which individual experiences can be explored against globally agreed benchmarks with respect to structures, cultures and institutions, and where routes to more effective or impactful implementation of rights across law, policy and practice can be outlined. While there is no human right to well-being, human rights treaties play an important role in setting out minimum core standards or conditions that contribute to overall well-being (Lundy, 2014). Although human rights frameworks have been increasingly used to consider children’s and disability rights, and the rights of deaf children with respect to education and language (Byrne, 2012, 2023; De Meulder & Murray, 2021), it is a novel approach for a human rights framework to be substantively applied to deaf children’s emotional wellbeing and to draw on the views of deaf children themselves in this context as rights-holders. Including emotional well-being. In this way, the article contributes to new knowledge internationally that can help both explain the lived experience of rights harms on emotional well-being and identify the solutions for preventing or managing threats to well-being for deaf children. “Rights harms” is used in this article by the authors to refer to the negative consequences that can arise when rights are not effectively implemented, are undermined, or when rights violations occur. The utility of a rights-based framework lies is in its ability to help us better understand and explain deaf children’s experiences in the context of state obligations and international benchmarks, thus shining a spotlight on human rights failures rather than deficit-based, individualised failures.

To demonstrate the usefulness of a rights-based framework (Lundy & McEvoy, 2012a, 2012b) for identifying and addressing the emotional well-being of deaf children, this study draws on a research project commissioned by a Health and Social Care Trust in Northern Ireland which sought to identify current and future service needs relating to the mental health and emotional well-being of deaf children. Firstly, we use evidence from the Northern Ireland Youth Wellbeing Study (NIYWS) to establish the prevalence of seven specific psychological conditions (e.g., Generalised Anxiety Disorder) of deaf children compared to their hearing peers. Secondly, we consider deaf children’s accounts of their emotional well-being and their perspectives on emotional well-being supports and services through a rights framing. The findings provide not only a lens for understanding the threats on deaf children’s emotional well-being but also a framework for promoting positive emotional well-being through the implementation of the rights of deaf children across law, policy and practice. Drawing on findings from our study we argue that deaf children experience a lack of inclusive time and space(s) across their lives in which they can meaningfully engage; and that they carry undue burden and responsibilities in supporting the implementation of their own rights despite their positioning as rights-holders; something that is not required of their hearing peers. We argue that, together, these factors contribute to the higher prevalence of mental health conditions they experience compared to their hearing peers.

The article begins by setting out the relevant literature in the field before outlining the relevance of human rights frameworks as a lens for emotional well-being. We then present our methodological approach before discussing our quantitative and qualitative findings. The latter is presented across three themes; firstly, the lack of inclusive time and space(s) in which deaf children can meaningfully engage; secondly, the undue burden and responsibilities on deaf children to implement their own rights; and thirdly; that a rights framework can act as a facilitator to better emotional well-being. We conclude that deaf children’s poorer mental health and well-being is evidence that their rights are not being adequately respected, protected or promoted. Through adopting a rights lens, we can not only see the issues but also identify clear solutions and tools in keeping with international law.

## Literature Review

### What we Know about Deaf Children and Well-Being.

Studies have generally found that deaf children have higher instances of mental health challenges than hearing peers (e.g., Li & Prevatt, 2010; Fellinger et al., 2009; NDCS, 2020; Remine & Brown, 2010). Research suggests between 11% and 63% of deaf children experience some form of emotional well-being difficulty (see NDCS, 2020). The wide variance in rates in these studies is likely due to who is reporting (deaf children, parents, teachers, clinicians) and what types of well-being are reported (e.g., emotional symptoms, conduct problems, hyperactivity inattention, peer relationship problems, negative social behaviour) (NDCS, 2020). Most studies, unfortunately, report only global measures of emotional well-being so little is known about rates related to specific disorders (NDCS, 2020). Overall, it has been estimated that deaf children have 1.5 times the prevalence of mental health struggles than hearing children (see Walker, 2013). In particular, research suggests that deaf children may experience higher levels of anxiety and depression (Fellinger et al., 2009). Furthermore, research has shown that deaf children experience higher risks of peer victimization, including bullying and sexual abuse (Kent, 2003; Kouwenberg et al., 2012). They are also more likely to experience a range of negative outcomes such as lower education attainment and higher rates of substance abuse as well as having less developed forms of coping strategies, self-esteem, and emotional competence (see Calderon et al., 2011). This has been primarily attributed to deaf children experiencing barriers in communication that can lead to exclusion and challenges in family and peer relationships (Fellinger et al., 2009; Wright, 2020). No known prior study has examined the prevalence of psychological conditions among deaf children compared to hearing children within Northern Ireland. This research addresses the gap by utilizing the Northern Ireland Youth Wellbeing Survey that captured these prevalences from a self-completed (when possible) questionnaire and included a representative sample of children and young people in Northern Ireland.

### Human Rights Frameworks as a Lens for Emotional Well-Being

Like children generally, deaf children are rights holders under the UNCRC (1989). The UNCRC contains a range of economic, social, cultural, civil and political entitlements covering almost all aspects of children’s lives (Fortin, 2005). In human rights treaty terms, deaf children are also identified as persons with disabilities and as having a particular cultural and linguistic identity and so are entitled to all the rights set out in the UNCRPD (2006) (Murray et al., 2018). The UNCRPD is a human rights treaty which sets out a range of rights with the aim to “promote, protect and ensure the full and equal enjoyment of all human rights and fundamental freedoms by all persons with disabilities, and to promote respect for their inherent dignity.” (Article 1 UNCRPD). Examples of such rights include equality and non-discrimination (Article 5), the right of children to express views (Article 7.3), awareness-raising (Article 8), accessibility (Article 9), access to justice (Article 13), freedom of expression, opinion and access to information (Article 21), and health (Article 25). There are a number of specific references to sign language across the UNCRPD including an obligation on States parties to: provide professional sign language interpreters, to facilitate accessibility to buildings and other facilities open to the public (Article 9); recognise and promote sign languages (Article 21); facilitate the learning of sign language and the promotion of the linguistic identity of the deaf community (Article 24); alongside an entitlement of deaf people to recognition and support of their specific cultural and linguistic identity, including sign languages and deaf culture (Article 30). Under Article 4 of the UNCRC and UNCRPD, States parties have committed to take “all appropriate legislative, administrative, and other measures for the implementation of the rights recognized in the present Convention.”

Despite this, there has been limited academic work that has sought to unpack what these rights mean in practice for deaf children or how outcomes for deaf children can be understood through a rights-based framework. Where work has been undertaken, this has predominantly related to the right to education for deaf children in relation to Article 24 UNCRPD (De Meulder & Murray, 2021; Murray et al., 2018; Snoddon & Underwood, 2017) rather than the broad spectrum of rights provisions.

Explicit recognition of deaf children as rights-holders is critical since it can disrupt the mainstream narrative of deaf children as children who are predominantly in need of protection or cure. Duty bearers, on the other hand, refers to those employed by the State, and who hold responsibility for implementing the obligations or “duties” set out in a treaty (Donnelly & Whelan, 2020). The importance of human rights treaties cannot be overstated. As Freeman (2010, p.52) has argued, the most fundamental of rights is the right to possess rights. Human rights treaties such as the UNCRC and UNCRPD provide a basis upon which individuals can claim entitlement and through which states that have ratified such treaties can be held accountable for their actions or lack of action through the monitoring process (Beitz, 2009; Lundy & McEvoy, 2012a). The “problem” in human rights terms, is the problem of the state (Quinn and Degener, 2002). An advantage of taking a rights-based approach in the context of deaf children, is that the State has a responsibility to tackle socially created obstacles to ensure full respect for the dignity of all deaf children and to ensure their full inclusion in society (as they do for children generally). This article therefore focuses primarily on factors external to the deaf child in exploring impacts on emotional well-being rather than assuming lower or poorer levels of emotional well-being is necessarily and inevitably a consequence of deafness. While some of these factors, such as communication barriers between deaf and hearing peers, have been highlighted in other literature it has not been done so with an explicit rights-based lens.

## Methods

This study applies a children’s rights-based approach developed by [institution] (Lundy & McEvoy, 2012a, 2012b). This adapts the United Nations Statement of Common Understanding of a Human Rights-Based Approach for research with children (Lundy & McEvoy, 2012a) to ensure that children’s rights are the focus of the study, that the methods adopted are rights-respecting and that children’s capacity to claim their rights is built and facilitated (see Lundy et al., 2021, O’Hagan & Byrne, 2023) Applying such a rights-based approach in our work means both applying human rights standards and emphasising the views of children and young people themselves using the Lundy model of participation (Lundy, 2007). This model presents the 4 interrelated concepts of Voice, Space, Audience and Influence to facilitate effective implementation of Article 12 of the UNCRC—the right of children to express their views and for those views to be given due weight. Whilst ordinarily, in our work, this includes involving children in the research in the form of an advisory group, in this instance establishing a young people’s advisory group was not possible due to the pandemic and project time pressures. Deaf children have, however, played a key role as research participants.

The study used a sequential mixed methodological approach. In the first stage, we drew on a survey to determine the prevalence of psychological conditions among deaf children and young people compared to hearing peers in Northern Ireland. Based on those, the second stage involved directly engaging with deaf children to investigate their experiences with emotional well-being and child and adolescent mental health services (CAMHS).^2^

### Stage 1: Quantitative Methods

The Northern Ireland Youth Wellbeing Study (NIYWS) 2019 was utilised as the first representative population-level survey for NI that samples children and young people on psychological wellbeing and captures information on hearing status. The survey sampled 3,074 children and young people between the ages of 2 to 19, including 67 participants reporting being deaf or having some hearing loss (hereto referred to as deaf for brevity). The age distribution roughly matched the census profile and was similar across deaf and hearing children with the deaf participants consisting of 23.9% aged 2–4, 29.9% aged 5–10, 17.9% aged 11–15, and 28.4% aged 16–19 whereas among hearing participants 20.8% were aged 2–4, 37.0% aged 5–10, 21.9% aged 11–15, and 20.3% aged 16–19. Children under the age of 11 had guardians complete the surveys, while young people 11 years old and older self-reported except when the young person was unable to due to a significant disability (n = 116). The survey was conducted in English and as in any household survey will under-represent subpopulations such as those living in refugee centres or residential homes although this likely a small portion of the total population (see Bunting et al., 2019). The survey was not made available in British or Irish Sign Language (BSL/ISL). We recognise this as a limitation.

Deafness was identified from questions on possible health conditions. The wording varied by age or who was answering the survey (guardian or self-reported). For example, self-reporting participants aged 11–15 were asked “Do any of these conditions or illnesses affect you in the following areas?” with the response option of “Hearing (for example deafness or partial hearing”) used to identify deaf participants.

Psychological conditions were derived from the Revised Children’s Anxiety and Depression Scale (RCADS: Chorpita et al., 2000). The scale consists of a 47 item questionnaire that indicates if children and young people are reaching clinically relevant levels of major depressive disorder, separation anxiety disorder, social phobia, generalised anxiety disorder, panic disorder, or obsessive compulsive disorder. The scale is standardised and age- adjusted; a child or young person is considered to have the condition if a certain threshold is reached. The scale has shown a good internal consistency, relatability and validity (Chorpita et al., 2000). Additionally, the study draws on the question “Please rate your quality of life on a scale of 1-5 from the 1 being the worst you can imagine to 5 being the best you can imagine”, which was completed by participants aged 11 to 19 years old.

Quantitative analysis consisted of cross tabulations with chi-square analysis determining if there are statistically significant differences in the six types of psychological conditions or any disorder, by hearing status. Furthermore, t-tests were conducted on the global measure on quality of life by hearing status. Notably due to the small sample size, these results should be interpreted with caution.

### Stage 2: Qualitative Methods

To determine what factors shape emotional well-being of deaf children, focus groups and semi-structured interviews were held with deaf participants ranging from 13–19. Participants were not asked directly about their own experiences due to the sensitive nature in a focus group context; nevertheless, many of them felt comfortable enough to voluntarily share personal aspects of their own emotional well-being.

Children and young people were recruited from sharing information via three mediums: through a regional deaf children’s non-governmental organisation, a national deaf children’s non-governmental organisation, and a deaf school in Northern Ireland. Two semi-structured interviews were conducted in May 2021. The first occurred when a focus group had only one available attendee (with a youth worker) and therefore shifted to an interview. The second interview was completed with a parent present. Additionally, three in-person focus groups were completed. In June 2021, two focus groups were held with deaf children: the first comprised six deaf children (three boys and three girls) aged 16–19 and the second consisted of three deaf children (three boys) aged 13–15. The third focus group was held in July 2021 and included six deaf children (4 boys and 2 girls) aged 12–16. Young people across the focus groups had a mix of communication preferences. Some were predominantly oral and relied on lipreading on sign supported English. Others had British Sign Language (BSL) as their first language. BSL interpreters were used in the focus groups with young people. The interpreters and young people were already familiar with each other. No Irish Sign Language (ISL) ISL interpreters were required.^3^ No interpreters were used or requested for the interviews.

Focus groups and semi-structured interviews were audio recorded with participant permission. After creating anonymised transcripts, data was coded and analysed thematically using Braun and Clarke’s (2006) six step approach (familiarisation with the data; generating initial codes; searching for themes; reviewing themes; defining and naming themes; and producing the report). The authors discussed and reviewed themes independently. No identifying labels are included to maintain anonymity of participants. There were no notable differences across the sample between girls and boys. Ethical approval for the study was obtained from the School of Social Sciences, Education and Social Work Ethics Committee at Queen’s University Belfast, Northern Ireland.

## Results

### Quantitative *Analysis*

The NIYWS was used to estimate the prevalence of psychological conditions among deaf children and young people and compared to hearing peers. Table 1 presents the percent distribution of having a psychological condition by hearing status. Among deaf participants, 20.9% had at least one psychological condition compared to only 12.3% of hearing participants. Among the psychological conditions examined, anxiety disorders stood out as having the highest levels of prevalence for deaf children and young people as well as the largest differences by hearing status. The highest prevalence for deaf participants was found in Separation Anxiety Disorder (16.1%), with high prevalence also in Anxiety (13.1%), and Generalised Anxiety Disorder (11.3%). Generalised Anxiety Disorder had the largest difference by hearing status with deaf participants having over 4.5 times greater prevalence than their hearing peers. Moreover, deaf children and young people had greater prevalence compared to their hearing peers of Panic Disorder (13.1% compared to 6.6%), Depression (11.3% to 4.9%), and OCD (8.2% compared to 3.0%). All of these differences were statistically significant using chi-square tests. Only Social Phobia Disorder was statistically non-significant with 4.9% of deaf participants having the condition compared to 3.7% of their hearing peers. Overall these findings highlight that deaf children and young people in Northern Ireland are experiencing high prevalence of psychological conditions (about 1 in 5), anxiety conditions appear to be acutely prevalent, and these conditions are far more common among deaf children and young people than hearing peers.

**Table 1:** Percent Distribution of having psychological conditions by deaf or has hearing loss

		Deaf or has hearing loss	Hearing	
Depression	Yes	11.3	4.9	c^2^(1) = 5.24, p = .022
	No	88.7	95.1	^*^
Total		100.0	100.0	
Anxiety	Yes	13.1	4.8	c^2^(1) = 8.66, p = .003
	No	86.9	95.2	^**^
Total		100.0	100.0	
Generalised Anxiety Disorder	Yes	11.3	2.5	c^2^(1) = 17.90, p = .000
	No	88.7	97.5	^***^
Total		100.0	100.0	
OCD	Yes	8.2	3.0	c^2^(1) = 5.37, p = .020
	No	91.8	97.0	^*^
Total		100.0	100.0	
Panic Disorder	Yes	13.1	6.6	c^2^(1) = 3.99, p = .046
	No	86.9	93.4	^*^
Total		100.0	100.0	
Separation Anxiety Disorder	Yes	16.1	4.9	c^2^(1) = 15.47, p = .000
	No	83.9	95.1	^***^
Total		100.0	100.0	
Social Phobia Disorder	Yes	4.9	3.7	c^2^(1) = .24, p = .628
	No	95.1	96.3	
Total		100.0	100.0	
Any disorder	Yes	20.9	12.3	c^2^(1) = 4.38, p = .036
	No	79.1	87.7	^*^
Total		100.0	100.0	

Note: ^*^ p < .05; ^**^p < .01; ^***^p < .001; to missing responses the sample size varies slightly across each condition. Total sample sizes for analysis on each condition with n = Deaf or has hear loss/n = Hearing: Depression 62/2871; Anxiety 61/2862; GAD 62/2875; OCD 61/2871; Panic Disorder 61/2867; SAD 62/2872; SPD 61/2867; Any disorder 67/3007

AltText: Table showing percent distribution of having psychological conditions by deaf or has hearing loss. Results show 20.9% had at least one psychological condition compared to only 12.3% of hearing participants. Among the psychological conditions examined, anxiety disorders stood out as having the highest levels of prevalence for deaf children and young people as well as the largest differences by hearing status

Additionally, Table 2 presents means comparisons for self-reports of quality of life among young people aged 11–19 by hearing status. Ranging 1 to 5 with high numbers representing higher quality of life, deaf young people had an average of 3.85 compared to 4.24 average from the other hearing young people. In other words, deaf young people reported statistically lower levels of quality of life than their hearing peers.

**Table 2:** Means comparison of quality of life among youth aged 11–19 by deaf or hearing loss status (score ranges 1 = Worst to 5 = Best)

	N	Mean	Std. Deviation	T-test	Cohen’s Effect Size
Deaf or has hearing loss	27	3.85	0.818	t(1161) = 277^^**^^	d = .45 medium effect
Hearing	1,136	4.24	0.733		

^^**^^p < .01

AltText – Table showing means comparison of quality of life among young people aged 11–19 by deaf or hearing loss status with scores ranging from 1 being worst and 5 being best. Deaf young people had an average of 3.85 compared to 4.24 average from the other hearing young people

### Qualitative Findings

In the sections that follow, we present findings from the data across three thematic areas to explore experiences and perceptions of emotional well-being among deaf children in Northern Ireland. We argue, firstly, that deaf children experience a lack of inclusive time and space(s) across their lives with which they can engage; and secondly, that they experience undue burden and responsibilities in relation to implementation of their rights. Together, these factors have negative impacts on their emotional well-being and may contribute to the higher prevalence of mental health conditions among deaf children compared to their hearing peers. Thirdly, we examine the role of a rights framework as a facilitator to “better” or positive well-being, drawing on young people’s views on solutions and ways forward. Ultimately, we argue that effective implementation of rights is a precondition to positive well-being; both in creating the conditions that can better support well-being on an equal basis with hearing children, and in giving deaf children the tools to challenge rights harms when they arise.

#### *Lack of inclusive time and space(s) across their lives in which they* can *meaningfully engage*

Time and space are central to our individual and social lives. Assumptions about time and space are shaped by the institutions and structures around us yet the manifestations of time and space tend to be invisible, taken for granted (McNeilly & Warwick, 2022) and grounded in embedded ideas about how they are typically used by individuals. The school day, for example, is mapped around timetables and structured routines of teaching, learning and task completion, while the healthcare system is predicated on rules around duration of appointments or interventions, targets and waiting lists. Time and space are not value free concepts. Crip time, for example denotes the need for time adjustments that a person might need to perform particular tasks (Ljuslinder et al., 2020; Samuels, 2017) while “deaf time” reflects the additional time that may be required to ensure effective communication among those who are deaf, as well as between those who are deaf and hearing (Snoddon and Underwood, 2017). We can identify similar assumptions about the use of space(s). As a socially constructed product (Lefebvre, 1991), O’Brien (2020) argues we must consider what sorts of spaces are produced, how and by whom. This can be seen in the idea of “deaf space” which reflects the “unique ways deaf people experience and modify space” (Kusters & Friedner, 2015; Per Koren & Haualand, 2014). It is important to consider not only physical spaces, but also cultural spaces and relational spaces.

We use the concept of “inclusive” time and space here to uncover and challenge dominant ways of being and doing and to consider the ways in which mainstream structures and institutions are based on exclusive, albeit often hidden, rules and principles. Inclusive time and space are important facilitators of rights. As noted earlier, Article 9 of the CRPD, for example, obliges States parties to take measures so that people with disabilities can access a range of spaces through environments, information and communication on an equal basis with others while Article 30 establishes entitlement to recognition and support of the specific cultural and linguistic identity of the deaf community, including sign languages and deaf culture This should include the provision of extra time and time flexibility. The data illustrates the lack of what we refer to here as “inclusive time and space(s)” for deaf children across various aspects of their lives, and the negative consequences of this for their emotional well-being. This lack is indicative of the ways in which the rights of deaf children are not being effectively implemented and the emotional harms that can arise out of these shortcomings. The examples that follow highlight both the subtle and not so subtle forms of rights harms and rights “wronging” (Kurki, 2018) that can occur when processes and spaces do not acknowledge or reflect the diverse needs of their users or recipients.

There was a consensus among the deaf children who took part in the study that their deaf peer group were much more likely to be anxious and to have poorer emotional well-being overall compared to hearing young people. In short, it was argued that participating in taken for-granted day to day activities was more difficult for deaf children. For example, one young deaf person said:

*It’s just hard because the common things that wouldn’t be a struggle to somebody else* can *be a massive thing*.

This was predominantly put down to communication challenges and a lack of deaf awareness among hearing people across services and facilities, contributing to challenges in accessing mainstream spaces not designed with deaf children in mind. There was also a perception among participants that more deaf people should be accessing emotional well-being services but were not due to communication barriers:

*I think the emotional help for a lot of deaf people...[they] should be getting more of it, [but] when, going into counselling if you need help for poor mental health, a lot of them aren’t deaf-aware. So,... having anxiety and depression, it’s hard to relate to the counsellor, and the counsellor struggles to help you out, because they don’t understand what it’s like not to hear and not feeling included because there’s a barrier between everyone*.

As this young person suggests, the lack of accessible or inclusive counselling spaces where deaf children can benefit on an equal basis as hearing children, combined with insufficient time to facilitate accessible communication, can exacerbate poor emotional well-being. This is despite the clear entitlements they have. In this instance we can see how not effectively implementing a deaf young person’s right to accessibility or appropriate healthcare (Article 9 and Article 25 CRPD) can have negative consequences for their mental health.

A young boy disclosed how they were accessing counselling via mainstream Child and Adolescent Mental Health Services (CAMHS) due to a difficult home situation. They did so with the assistance of a sign language interpreter. However, they did not find this helpful and said they did not want to share what was going on with CAMHS as they did not want the issues to be “on record” or to share personal information via a third-party interpreter. Instead, they expressed preference to communicate directly in sign language to a mental health professional so they did not have to go through a third person. This service was not available to them. Here, even where a deaf young person did engage with seemingly available and accessible specialist mental health services, they still felt disadvantaged and that they were not accessing this service on the same basis with others. Whilst in theory the availability of an interpreter would seem to indicate accessibility and inclusion, and thus align with the CRPD, in practice this would appear to be an example of minimal accessibility.

Similarly, a young boy disclosed how he was meant to see a deaf counsellor and was happy with this but ended up seeing a hearing counsellor who could not use sign language instead due to a long waiting list. He felt frustrated that his preferences were not “listened to.” One young person spoke of the negative message conveyed by these long waiting lists. Having been on a waiting list to see a counsellor for 18 months at the time of meeting, they said this made them feel “*what is the point? They don’t care about deaf problems.”* These examples can be linked to a concept of inclusive and exclusive relational space; that is, the ways we interact, communicate and relate to each other, how we are represented and understood in these spaces and our sense of belonging therein. A focus group of young people in the study discussed how they felt unable to talk to their family and did not have anyone in their lives they felt understood them and that they could trust enough to share how they felt.

The persistence of bullying was emphasised among participants as a key factor contributing to poorer emotional well-being compared to hearing children. For example, one girl shared how a (hearing) child in school took her hearing aid from her and put it in a bin while others spoke of being “picked on” because of their speech. The extent of bullying experienced by deaf children across settings (for example, Cheng et al., 2019; Kent, 2003; Pinquart & Pfeiffer, 2014) is indicative of the emotional and rights harms that can arise when deaf children’s experiences of their rights fall short of minimum entitlements that should be provided by States. Protecting, promoting and fulfilling deaf children’s human rights in ways that, for example, address bullying or negative stereotypes, and contribute to more welcoming and inclusive spaces can reduce potentially negative influences on emotional well-being. Other deaf children found it difficult to make friendships with hearing people in their local community. Despite making plans and taking part in activities such as football, they found that hearing children also taking part spoke too fast and did not take time to explain things with the result that they felt “left out” and isolated. This is an example of both the lack of inclusive relational spaces as well as the lack of inclusive time for accessible interactions to take place. One deaf boy discussed how he found boxing really helped him deal with frustrations and anxieties on the one hand but, because it was a hearing boxing club, it brought its own separate anxieties as he found it difficult to communicate with others who attended the club.

The experiences of deaf children accessing mainstream services and facilities in their local communities contrasted with the experiences of the minority of deaf children in the study who had friendships with other deaf children either through school or local deaf organisations and who said they found it easier to be with deaf people, that they could “relax” and feel more included. Conversely, they experienced positive benefits from inclusive physical, cultural and relational spaces with other deaf young people.

The importance of inclusive time extends to the classroom. All participants emphasised that school life was exhausting and said they found it difficult to concentrate. One boy said that he did not see any friends outside school, and he preferred to be alone in the evenings as he felt so exhausted from attending mainstream school. Listening fatigue and struggles to concentrate in class whether using a sign language interpreter or lipreading and/or using FM equipment was a recurring theme among deaf children in the study. This had a subsequent impact on how they felt:

*Because I know when somebody says after three to four points, and if it’s really intense conversation and I don’t fully get it, I’m like my brain goes dead. I just* can*’t focus, I then get very emotional and then I’m just a big mess.*

The above examples, taken together, illustrate the consequences of the lack of inclusive time and space(s) on the emotional well-being of deaf children, despite the obligations which require active measures to be taken.

#### Undue burden and responsibility on deaf children to implement their own rights

In human rights terms it is the State party and those employed by the State party who are duty-bearers and upon whom obligations to promote, protect and fulfil rights fall (Donnelly & Whelan, 2020). Individuals, and in this case, deaf children, are rights-holders. They do not need to actively do anything to claim their rights (Donnelly & Whelan, 2020). The “burden” of implementing rights fall on adult professionals whether across government departments or public bodies. Our data suggests that deaf children are in a unique position as rights-holders in that they appear to also become indirect and unofficial duty bearers through the need in particular cases to take on extra responsibility for either implementing their own rights or facilitating an environment for professionals to be able to implement their rights more effectively. In short, the data suggests that deaf children carry extra or undue burden and responsibilities in supporting the implementation of their own rights despite their positioning as rights-holders; something that is not required of their hearing peers. The burden goes beyond merely standing up for their rights but taking measures or actions that would be expected of adult duty-bearers and additional pressures of navigating structures and institutions. Carrying such burden and responsibility can have negative consequences for the emotional well-being of deaf children.

Some deaf children in the study had experience of accessing CAMHS. For example, a deaf girl shared her experience of accessing support through mainstream CAMHS services due to high levels of anxiety. She told us:

*I’d got help with my anxiety,... I had to leave class because it was just a mess. But yeah, yeah it’s just learning how to use them. I got help through* [NGO] *and just CAMHS as well, they helped. And it’s just having, knowing that certain things are normal, certain things I* can *deal with and certain things that I need help with. Like it’s knowing that when you’re about to vomit it’s not a normal thing.*

She managed to access support during the first pandemic lockdown in 2020. However, the counsellor did not appear to be deaf aware, and she had to “teach” them how best to communicate with her before being able to proceed with the counselling itself. Doing so can compound already poor emotional well-being:

*I had to kind of teach them a little bit, because they didn’t know how to approach me and it made me feel a bit iffy at the beginning. They did do well and, wearing masks at that stage, when masks were a thing, I just wore the mask and then they took it off, and it meant that I could communicate with them.*

While being able to “teach” the mental health professional meant the child was able to tailor their support to a certain extent, having to do so before being able to effectively access support places additional pressures and responsibility on the child. It is important to remember that deaf children are rights-holders, not duty-bearers, and there is an obligation on healthcare professionals to undergo relevant training.

There were examples of deaf young people seeking to develop their independence and assertiveness as they made the transition to young adulthood but found themselves also having to develop strategies to “manage” challenges or barriers to developing their independence and in maintaining their well-being, despite theoretically having the right to full inclusion in the community (Article 19 CRPD). A girl explained how her speech was perceived to be “very soft” and that others may have found it difficult to hear her, leaving her feeling very embarrassed and anxious by that each time she went out. Others spoke of the internalised frustration they had if they mispronounced words in public and that this was embarrassing for them. The emphasis on speech in these instances was despite their preferred use of sign language. Three girls from a “special” school shared how they tended to go to a specific shop assistant, and avoid all other shop assistants, in a local shop after school who knew a little bit of sign language and was deaf aware and that this helped them to feel more confident and at ease. These examples speak to the subtle ways in which deaf children pre-empt and feel pressure to create or “manage” otherwise inaccessible situations.

For those participants who attended mainstream school, there was a sense that they needed to be more confident in certain situations to highlight when support needs were not being met or equipment was not being used properly. They felt the onus was on them to point this out, perhaps in front of a large class of children in a mainstream environment. This is despite the responsibility on teachers as duty-bearers to ensure they use equipment appropriately.

*When using FM in class I find sometimes teachers forget how to use it, and maybe have it facing the opposite way round so it’s making it so I* can*’t hear them..., it’s hard to find the courage then to turn round and be like, in the middle of class, and it’s just, it makes you feel low because you* can*’t get your, you know, the things you want to say. And then it’s when you’re struggling with certain things and you’re getting overwhelmed it’s hard to approach a teacher when they’re already unapproachable. If they’re in a bad mood, if you’ve interrupted them in the class, and if you’re struggling with something it’s really, really hard to address that you need that extra help in a certain area.*

Deaf children across the study discussed the skills they felt was important for them in addressing barriers and subsequently alleviating anxieties and frustrations:

*I think, you know, having the confidence then to turn round and say to somebody, “look you’re not doing it correctly” or, you know? And I think as well when needing the help, it’s knowing how to get it. It’s trying then to get the right help.*

In an ideal world deaf children would not have to develop additional skills to navigate an inaccessible society. While the development of such skills is important in empowering children and supporting them to stand up for their rights, there is a fine line between standing up for rights and internalising responsibility for promoting their own rights, the undue burden of doing so, and the detrimental impacts on emotional well-being this can have.

#### A rights framework as a facilitator of positive emotional well-being

While, as noted earlier, there is no standalone right to good well-being, there are rights to various conditions that can support well-being. In this way, a rights framework can act as a critical facilitator of positive well-being through shaping accessible and inclusive environments and reducing rights harms or wronging. While doing so will not eradicate all factors impacting on a young person’s well-being, it may help to address the higher prevalence of mental health conditions among deaf children compared to their hearing peers where these are related to environmental, cultural and relational factors. This final theme, therefore, centres on ways forward and solutions as highlighted by participants, using rights framing.

Deaf children in the study emphasised the importance of engagement with deaf peers in supporting well-being, particularly among those who found themselves to be the only deaf student in a mainstream school. This underscores the importance of the entitlement of deaf children to recognition and support of their specific cultural and linguistic identity (Article 30.4 CRPD). Facilitating contact with deaf peers either through funding deaf organisations or deaf youth groups or setting up specific programmes are some examples of how this right could be implemented, subsequently supporting deaf children’s emotional well-being. Accessing peer support through an external organisation was viewed by participants as being vital. It was argued that this would create a sense of community that deaf children could draw on and engage in if they were struggling with their emotional well-being but did not necessarily need to access CAMHS or a counselling support:

*I also think it would be perfect if they had a group of people that felt the same way and being able to relate with some people. Because then you kind of feel that you’re not entirely on your own and that there’s other people in the same boat and that you* can *talk to them as well, if maybe you don’t need the counsellor but you just need like a friendly chat with a friend.*

Article 9 of the CRPD obliges States to ensure accessible services and facilities are provided in both urban and rural areas. However, some children expressed concern that existing support was not necessarily close to where they lived and meant they were only able to attend social activities on an irregular basis. Other participants emphasised the need to use online facilities that their peer group were already familiar with and would perhaps feel comfortable using as a way of expressing emotions. Some deaf children acknowledged that child focused helplines were not necessarily accessible for those who were deaf and that they were effectively excluded from accessing support.

*I think they should have like a website or a WhatsApp group for people, and if they are feeling low if they could just have that chance just to say to people about how they feel, through text message. And even if it was that they could FaceTime with somebody if they were really struggling with something, just that reassurance would make a massive difference. Because all of those calls, stuff, they leave people [out] who* can*’t go on the phone. And yeah sometimes people* can*’t put into words how they feel, but if they message it, it becomes easier to say if they’re feeling low, if they’re struggling with friends, if they’re struggling in school, or they’re just needing someone to listen.*

Article 9 CRPD extends to the provision of accessible information and communication technologies with the CRPD committee (2014) noting that “Without access to information and communication, enjoyment of freedom of thought and expression and many other basic rights and freedoms for persons with disabilities may be seriously undermined and restricted” (para 21). One young person had availed of a text messaging facility belonging to a mainstream helpline. She had found this helpful but was somewhat frustrated at the length of time it took to get a response to her message each time. Participants in the study said they would be more likely to access support if that source of support was deaf aware, deaf specific or part of a broader deaf network.

Lots of ideas were shared about the type of emotional well-being support that could be offered, both in terms of structure and content. In one group discussion about what services they would like, a boy said he would have a “land” just for deaf people who would design all services. While this was somewhat tongue in cheek, it relates to a broader idea of feeling part of a community, deaf identity, and being able to access services without having to think about barriers. Deaf children across the study discussed the skills they felt was important for them in addressing barriers and subsequently alleviating anxieties and frustrations. For example, support with building confidence was seen as an essential skill in lots of contexts:

*I think, you know, having the confidence then to turn round and say to somebody, “look you’re not doing it correctly” or, you know? And I think as well when needing the help, it’s knowing how to get it. It’s trying then to get the right help.*

Developing a range of supportive techniques was presented as key. This was not just about general techniques to reduce anxiety or stress but understanding the need for deaf specific techniques tailored to situations. For example:

*I just think very much knowing how to cope, because I would have quite bad anxiety and I would have panic attacks, those kinds of things. And by having the techniques that I* can *use, like when I’m going to the shop maybe putting my hearing aids down just a little bit so that I’m not getting overwhelmed by all the noises and people. And having an FM system when it’s one-to-one with my mum if we’re in the shops, so I’m only focusing on one voice rather than a hundred, with everyone. It’s having the techniques, like the five minute rule, just kind of things that, even having, like being able to fiddle with a pen, being able to express yourself.*

From a rights perspective, all children have a right to be supported in their physical, mental, spiritual and cultural development (Article 5 UNCRC, 1989). Support in identifying and learning appropriate coping strategies aligns with this, and, in the context of this study, could be understood as inclusive development; that is, the development of strategies that allow deaf children to reclaim agency across challenging contexts. Some deaf children recognised that there was a variety of resources online that were available, and that increasing attention was being paid to these. However, it was argued that these did not always speak to their situation and were rarely deaf specific and age appropriate:

*All the YouTube videos aren’t geared to anything with deafness. So they Google up something and it’s for general, hearing, everybody who’s hearing or doesn’t have a disability, it’s all geared to people like that. But if it was more geared to people that* can *use certain techniques, like using the hearing aid, putting it down a little bit in certain situations, having time set out during the day to take them off, to relax properly, and just having more time I think.*

There was some discussion around whether counsellors or emotional well-being professionals should be deaf themselves. There was a clear preference across the focus groups overall for a deaf counsellor or deaf professional associated with any service, and that this would encourage them to share issues or worries they might have. Suggestions included supporting or training more deaf people to become counsellors. For some young people it was more important that a service be deaf specific, have expertise on deaf issues or be properly deaf aware:

*I don’t think it matters if they’re hearing or deaf or if they’re a counsellor or somebody who you see on a daily basis. I just think what would make more sense, if people who are in one of those things were deaf-aware and could have an* idea *of what it would be like. So then they* can *understand that bit better of how somebody feels when they’re socially isolated from everyone.*

Deaf awareness was highlighted time and time again, along with the importance of everyone learning basic sign language so they could communicate with deaf children. Another young person suggested that, as well as being deaf aware generally, it was important to be aware that deaf people are different from each other and have different needs, including different communication needs. This aligns with Article 8 of the CRPD on awareness-raising which obliges States to take “immediate, effective and appropriate” measures to raise awareness regarding people with disabilities—and in this context, deaf children—to “foster respect” for their rights and dignity, to combat stereotypes, and to promote awareness of their capacities and capabilities (Article 8.1 CRPD).

In cases where a child was a sign language user it was argued that the professional or counsellor should also be proficient in sign language rather than having to go through a sign language interpreter who is effectively a third party. This brought up concerns around privacy and information protection, particularly given the relatively small size of the deaf population in Northern Ireland and where sign language interpreters may be known on a personal as well as professional basis. There was concern that having to use a sign language interpreter rather than communicating directly would put some deaf children off from engaging with emotional well-being and mental health services:

*[I]nstead of having to get somebody else to come in it should be done through the person, so it’s a more one-to-one, rather than having it through two other people, which then makes it more awkward, and you feel that you have to explain the situation to two people and hope that they don’t, you know, go home and say it to somebody else.*

In addition to creating conditions for inclusive and accessible time and space, a rights framework extends to individuals learning about their rights. For deaf children, learning about their human rights provides the cultural capital needed to call rights (in)actions into question, to challenge rights harms and wronging as well as undue burdens and responsibilities. Explicit recognition of deaf children as rights-holders is critical since it disrupts the mainstream or traditional narrative of being in need of protection or cure.

## Discussion and Conclusion

This paper aimed to explore the emotional well-being of deaf children from a rights perspective, drawing on both the UNCRC and UNCRPD. It explicitly situates deaf children as rights-holders with fundamental entitlements. Our secondary analysis of the NI Youth Wellbeing Survey illustrates that deaf children are more likely to have one or more mental health condition compared to their hearing peers. The views of deaf children on emotional well-being, the services offered and needed improvements are core to our study. Our findings uncover the ways in which individual emotional well-being can be directly or indirectly impacted by surrounding environmental, cultural and relational factors. These factors can be understood as evidence of the ways in deaf children’s rights are not being effectively implemented, and which can give rise to emotional harms.

Specifically, we argue that deaf children experience a lack of inclusive time and space(s) in which they can meaningfully engage, and that they carry undue burden and responsibilities in supporting the implementation of their own rights despite their positioning as rights-holders; something that is not required of their hearing peers. The rights harms and wronging that deaf children experience, as evidenced through this article, are indicative of the subtle and often hidden forms of discrimination that can persist. Indeed, the subtleties of taken for granted and exclusive or “hearing” ways of beings and doing can be all the more powerful and damaging when their social construct is normalised or silenced.

The children in our study made some suggestions around support in identifying and developing coping strategies or tools. While the term “resilience” can have negative, individualistic connotations (Traynor, 2017), we suggest that deaf-specific human rights education has a key role to play in supporting deaf children and young people to stand up for their rights, and to act as human rights defenders (Lundy et al., 2023), whether they choose to use the term or identify themselves as such. This reflects findings from Young et al. (2023) that deaf young people with greater self-determination (skills related to feeling empowered to navigating one’s life) related to higher levels of wellbeing.

Our study has a number of strengths including producing the first estimate of psychological conditions prevalence by hearing status among children and young people in Northern Ireland using a representative sample. Furthermore, this study is novel in applying a rights framework to understanding the threats to emotional wellbeing among deaf children internationally. Moreover, our data was produced primarily by directly engaging with deaf children to get their perspectives on emotional health. There are some limitations to the study. The scale to measure the psychological conditions was designed for self-completion of child/youth ages 8–18. Further versions for parents to complete were created for 4 years and older; however, it is important to note that NIYWS included children 2–3 in the analysis.

Additionally, this survey was not designed specifically for deaf children and therefore the number of deaf respondents in the sample was relatively small, which limited the complexity of the analysis.^4^ Moreover, as the survey was not designed specifically for deaf children and young people, it did not distinguish between deaf or hard of hearing, preferred communication method, or other characteristics that can be useful in understanding the experiences and outcomes of deaf people. Future studies could consider having a survey designed specifically for research deaf children and young people that could provide more nuanced quantitative findings and should be made available in signed languages. Conducting the qualitative component during the pandemic meant that there were restrictions and challenges in recruiting for the study in light of social distancing and lockdowns. We acknowledge that this is a small sample of children and skewed towards the older age range. This does not diminish the value of those who took part in the study—as Article 7.3 affirms, all children have the right to express their views and for these views to be given due weight.

The utility of a rights-based framework lies is in its ability to help us better understand and explain deaf children’s experiences in the context of state obligations and international benchmarks, thus shining a spotlight on human rights failures rather than deficit-based, individualised failures. The article contributes to new knowledge internationally around understanding deaf children’s emotional well-being by recognising the impacts of rights harms and wronging, however subtle. We argue that deaf children’s poorer mental health and well-being is evidence that their rights are not being adequately respected, protected or promoted. For example, if state obligations to ensure accessibility of services were met, deaf children may feel less embarrassed, stressed or socially isolated. Through adopting a rights lens, we can not only see the issues but also identify clear solutions and tools in keeping with international law. Many of the solutions identified are measures which states are already obliged to put in place. The evidence base here reinforces the need for these measures to be taken.

## Endnotes

1 We use the term “deaf children” throughout the paper to encompass deaf children and young people aged 0–18. We use small “d” deaf in this paper to reflect the diverse experiences of the children and young people who took part in our study.

2 CAMHS is a terminology employed by the NHS and other organisations in the UK to refer to specialised services aimed at the mental health for young people. Although it can be used broadly across organisations, interviewees in the study are mainly using the term in reference to supports given or wanted from the Health and Social Care Trusts in Northern Ireland.

3 Northern Ireland has two signed languages—British Sign Language (BSL) and Irish Sign Language (ISL). According to the Department of Health, there are approximately 8,000 sign language users in Northern Ireland. The Continuous Household Survey in 2013/14 reported that 9% of adults in Northern Ireland could use a sign language, with 8% using BSL and 1% using ISL.

4 Chi-square tests require expected cell size of n = 5 or larger, due to the small cell size only any disorder meet the normal threshold and therefore should be interpreted with caution. A Yates’ continuity correction is sometimes applied to 2x2 chi-square distributions; however it is generally considered to produce too conservative results and was not reported in Table 3. If Yates’ correction is applied, depression, anxiety, generalised anxiety disorder, and separation anxiety disorder remain statistically significant and therefore may be more robust findings compared to OCD, panic disorder and any disorder.
